# “Selling” Value: The Influence of Language on Willingness-to-Accept

**DOI:** 10.1371/journal.pone.0120292

**Published:** 2015-03-30

**Authors:** Kirk F. Manson, Ifat Levy

**Affiliations:** 1 Section of Comparative Medicine, Yale School of Medicine, New Haven, CT, United States of America; 2 Department of Neurobiology, Yale School of Medicine, New Haven, CT, United States of America; The University of Kansas Medical Center, UNITED STATES

## Abstract

In behavioral economics, the “endowment effect” describes the robust finding that prices people are willing to accept (WTA) for a good exceed prices people are willing to pay (WTP) for the same good. The increase in WTA values is often explained by the sellers’ negative hedonic response to losing their item. Recent studies, however, show that subtle cues may change participants’ perspective, influencing their valuations. We hypothesized that implicit connotations of instructional language may be one of those cues. To test this hypothesis we manipulated the wording of instructions in two conditions: in the Sell condition, subjects were endowed with a set of pens and asked to select an amount of money for which they would sell the pens back and in the Take condition, subjects were endowed with the pens and asked to select an amount of money they would take for the pens. Participants in each condition also estimated the market value of the pens. Consistent with our hypothesis, WTA in the Sell condition was higher than in the Take condition, though there were no differences in market values between conditions. These findings show that instructional language does influence participant valuations. Furthermore, we suggest that those being asked to “sell” use their market estimations as the salient reference point in the transaction.

## Introduction

In experimental market exchanges, prices sellers are willing to accept (WTA) for a good typically exceed prices that, as buyers, they are willing to pay (WTP) for the same good. Thaler (1980) termed this trend “the endowment effect” and it has since been studied extensively [1]. Many studies proposed loss aversion [2], or the increased weighting of losses compared to gains, as the driving force behind the increased WTA prices. These studies suggested that the pain from losing an item looms larger than the reward from acquiring the same item. Thus, sellers will increase the price they demand for parting with their good [3–7].

Although the ultimate motivation for the increased WTA may be intrinsically rich, like loss aversion, the more proximate mechanism(s) may not be. Contextual cues may make some aspects of a transaction more salient than others and influence participants’ valuations. For example, prices provided with goods (e.g price tags) act as indicators of the objective market values of the goods. Weaver and Frederick (2012) argued that sellers evaluate potential trades with respect to these objective market prices and these prices typically exceed the sellers’ own subjective valuation of the good [8]. Furthermore, to avoid taking a “bad deal” sellers raise their WTA prices to be more in line with the market price. Consistent with this theory, in a series of studies Weaver and Frederick have shown that WTA amounts are considerably reduced when market prices align more closely to participants’ subjective values, compared to when market prices largely exceed these subjective values. These findings dovetail with findings by Simonson and Drolet (2004), who showed that when participants are motivated to sell they reference the objective market values in deriving their WTA values [9].

Variations in the wording of the instructions used in different market paradigms can also alter participants’ perceptions of the task. These variations, which can be easily overlooked by an experimenter, may convey unintended information to the participants and bias their valuations. Value estimates are influenced, for example, by whether the demand for a transaction is perceived as coming from the seller or from the buyer [10]. Similarly, short phrases in the instructions of a trading paradigm can inadvertently signal differences in the nature of the trade (e.g. whether the endowed good was perceived as a gift) and explain trading asymmetries better than would be predicted by the loss aversion account [11].

Experimenters do not necessarily need to provide explicit information to change participants’ perceptions. Subtle, and sometimes imperceptible, cues can prime traits or goals associated with specific affective or cognitive constructs, such as prestige, thriftiness, or impatience, in decision-making tasks (e.g. [12–13]). Once the constructs are activated, participants tend to make choices consistent with the associated traits or goals. Chartrand and colleagues (2005), for example, showed that participants primed with thriftiness chose less expensive items [12] (for a review and discussion on [non]conscious influences of consumer behavior, see [14–15]).

In the current study we aimed to extend these previous findings in two important ways. First, we hypothesized that the exact language used to instruct participants in market paradigms may affect the reference processes illuminated by Weaver and Frederick (2012), by putting sellers in a “selling state of mind” [16]. One previous study did observe a valuation difference between conditions even in the absence of the words “sell” or “buy” [17]. That study, however, lacked comparison conditions in which these words were used, and therefore the effect of the wording manipulation is still unclear. Second, objective market prices are not always explicitly available in day-to-day transactions. Therefore, we investigated whether participants have a general presumption of the market price of a transaction good and how those market prices act as references when sellers establish WTA prices.

## Method

### Participants

The study was approved by Yale University's Human Investigation Committee. One hundred and sixty-eight participants (83 female, ages: 18–47, mean age: 21.4 ± 4) recruited at Yale University signed an informed consent. Participants knew upon recruitment they would receive $10 as a show-up fee and an additional reward for their participation.

### Study 1

Ninety-eight participants (48 female, ages: 18–47, mean age: 23 ± 5) were randomly assigned to either a Sell (n = 53) or Take condition (n = 45), in which both the ownership of a good and instructional wording (i.e the frame of the task) were manipulated. Participants were endowed with a pack of 8 Uniball multicolor gel pens with a retail value of $8.96. They then indicated their market estimation of the pens, how much they liked them and how much they were in need of the money at the time. Participants in the *Sell* condition received the pack of pens at the outset of the task as their reward for participation. They then received a survey with the market prices and rating questions on one side, and a transaction scenario on the other; which side they saw first was alternated throughout the study. Once they completed the front side, the experimenter told them to turn over the survey and complete the rest. The transaction scenario reiterated that they owned the pens and then asked them to pick a bottom line price from a list of dollar amounts (i.e. their WTA), ranging from $0.50 - $14.00, in increments of $0.50, for which they would sell the pens back. The instructions also informed them that it was important to indicate the actual value that the pens were worth to them, as the experimenter was going to randomly produce an offer for the pens from the same list of dollar amounts. If the offer was below their bottom line, they would keep the pens; if the offer was at or above their bottom line, they would sell the pens back [18]. The participants read these instructions and repeated them aloud in their own words prior to choosing. If they seemed to not understand the task, the experimenter went back through the instructions and clarified as needed. They then made a subjective price selection and the outcome was actualized.

The procedure for the Take condition was nearly identical to the Sell condition, except the word “sell” was omitted from the market scenario. Instead, the instructions asked participants to pick an amount of money they “would *take for* the pens”. For full instructions to each condition, see S1 Instructions.

### Study 2: Replication

Sixty-two participants (35 female, ages: 18–28, mean age: 20 ± 3) were randomly assigned to either a Sell (n = 31) or Take condition (n = 31). The procedure was nearly identical to the previous procedure, save for two changes: 1) we used the same set of pens as in Study 1, however the retail price of the pens had increased to $9.41 at the time of Study 2. 2) Because the use of a scale of numbers may introduce an additional reference point [8], we omitted this scale from the transaction survey and allowed participants to freely write in a WTA price.

### Data Analysis and Hypothesis

We opted to use non-parametric techniques to analyze the data due to skewness that violated the assumptions of standard parametric analysis. We made pair-wise comparisons using a Mann-Whitney U test. The null hypothesis in this test is that the medians in each condition came from the same distribution. If strategic connotations of the word “sell” influence valuation beyond endowment status, then we should expect median valuations in the Sell condition to be higher than those in the Take condition. Otherwise, we should see similar median valuations in the Sell and Take conditions. Additionally, if the influence of instructional language extends beyond subjective value to participants’ general perceptions of the pen set’s value, then we should see differences in market estimations consistent with the aforementioned hypothesis.

## Results

### Study 1

Participants rated how much they liked the pen set and how much they were in need of money at the moment on a Likert scale from 1–5 (1 = not at all, 5 = very much, see S1 Dataset for full dataset). A Mann-Whitney U test indicated no difference in how much the participants liked the pens (Sell condition median = 4.00, Take condition median = 4.00, U = 927.5, p = 0.10). Additionally, there was no difference in economic need (Sell condition median = 3.00, Take condition median = 3.00, U = 832.5, p = 0.41).

**Fig 1:**
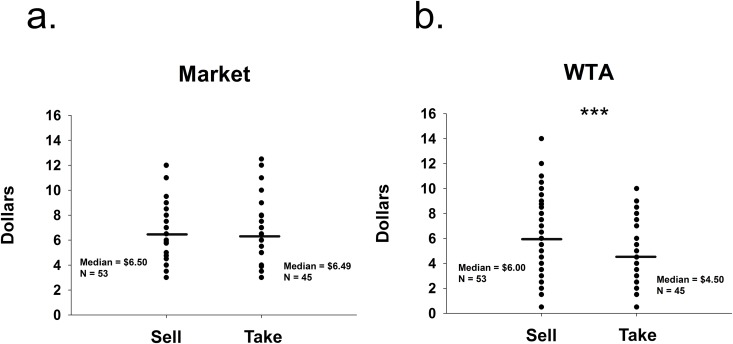
Study 1. a) Study 1 distribution of participants’ median market estimations (Market) of the pens. b) Study 1 distribution of willingness-to-accept (WTA) median values indicated as the participants’ bottom lines to either sell back their pens (Sell) or take money for their pens (Take); *** significant at p = 0.001, (Mann-Whitney U test).

Participants estimated the market value of the pens. A Mann-Whitney U test indicated no difference in market estimations between conditions (Sell condition median = $6.50, Take condition median = $6.49, U = 1174.50 p = 0.90, Fig. 1A). Whether participants were asked for market estimations before or after giving their WTA had no effect on prices (Sell condition: U = 326.50, p = 0.66; Take condition: U = 232.00, p = 0.80).

The main measure of interest was participants’ bottom line prices, i.e. their willingness to pay (WTA). Pairwise comparisons using a Mann-Whitney U test revealed the Sell condition WTA values were higher than those of the Take condition (Sell condition median = $6.00, Take condition median = $4.50, U = 718.50, p = 0.001; Fig. 1B). Whether participants were asked for WTA prices before or after giving their market estimations had no effect on WTA prices (Sell condition: U = 300.00, p = 0.36; Take condition: U = 205.00, p = 0.37).

Thus, in Study 1 we show that participants who were specifically asked “to sell” a good had higher WTA values compared to participants who were asked how much they would “take for” their good. We also show that participants’ market estimations of the good do not differ between conditions, suggesting the effect does not extend to participants’ general perceptions of the good’s value.

These surprising findings may seem at odds with one of the prominent explanations for the endowment effect. Several studies [1, 2, 17] suggested that loss aversion, or the pain of losing an endowed item, is the sole factor driving WTA values to be higher than WTP values. Loss aversion, however, cannot account for our results, since in our study participants in both the Sell condition and the Take condition were asked to give up a good with which they had been endowed. To confirm the results we therefore conducted a second study with an independent sample of participants.

### Study 2: Replication

Participants rated how much they liked the pen set and how much they were in need of money on a Likert scale from 1–5 (1 = not at all, 5 = very much). Pairwise comparisons using a Mann-Whitney U test revealed no difference in how much the participants liked the pens (Sell condition median = 4.00, Take condition median = 4.00, U = 473.00, p = 0.90) or in economic need (Sell condition median = 3.00, Take condition median = 2.00, U = 450.00 p = 0.66).

**Fig 2:**
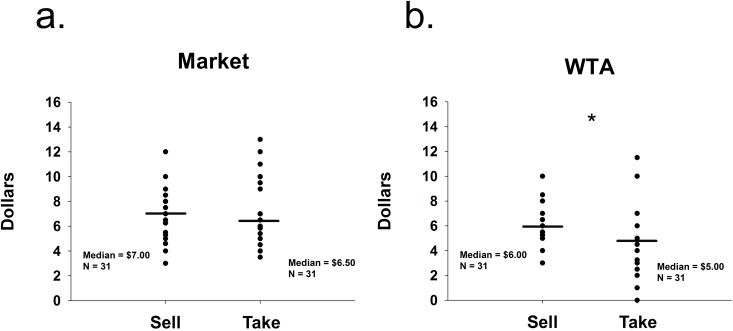
Study 2. a) Study 2 distribution of participants’ median market estimations (Market). b) Study 2 distribution of willingness-to-accept (WTA) median values indicated as the participants’ bottom lines to either sell back their pens (Sell) or take money for their pens (Take); * significant at p < 0.05, (Mann-Whitney U test).

We examined participants’ WTA values and market estimations in two conditions. If the word “sell” influenced participants to raise their prices, then we should observe higher WTA prices for the Sell condition compared to the Take condition. Pairwise comparisons using a Mann-Whitney U test revealed that market estimations were not significantly different between conditions (Sell condition median = $7.00, Take condition median = $6.50, U = 429.00, p = 0.47, Fig. 2A). Conversely, as in Study 1, the Mann-Whitney U test revealed that WTA values in the Sell condition were significantly higher than in the Take condition (Sell condition median = $6.00, Take condition median = $5.00, U = 323.00, p = 0.03, Fig. 2B). Again, the order in which participants received the questions had no effect on WTA (Sell condition: U = 103.00, p = 0.50; Take condition: U = 115.00, p = 0.87) or market estimations (Sell condition: U = 120.00, p = 1.00; Take condition: U = 114.00, p = 0.84). Thus, these results replicated the findings of Study 1.

### Relationship of WTA to Market Estimations

Studies 1 and 2 provide insight into how language affects participants’ valuations. The nature of the relationship between WTA values and market estimations, however, is still unclear even in light of these results. Individually, our two studies do not provide enough statistical power to allow for proper correlational analysis to answer how WTA values relate to market estimations.

To increase statistical power we therefore combined the two datasets. We first verified that there was no significant difference between the results of studies 1 and 2. A Mann-Whitney U test indicated neither a difference between the two Sell conditions (median Sell WTA 1 = $6.00, median Sell WTA 2 = $6.00, U = 756.00, p = 0.54; median Sell Market 1 = $6.50, median Sell Market 2 = $7.00, U = 720.00, p = 0.35) nor a difference between the two Take conditions (median Take WTA 1 = $4.50, median Take WTA 2 = $5.00, U = 636.00, p = 0.51; median Take Market 1 = $6.49, median Take Market 2 = $6.50, U = 669.00, p = 0.76), supporting the appropriateness in combining the two datasets.

**Fig 3:**
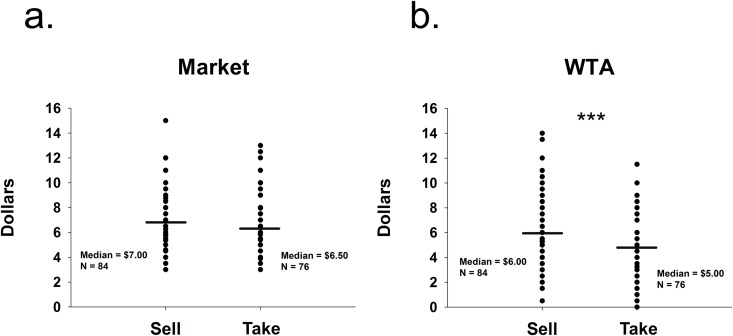
Combined Study 1 and Study 2. a) Distribution of combined median market estimations (Study 1 and Study 2). b) Distribution of combined willingness-to-accept (WTA) median values. *** significant at p < 0.001, (Mann-Whitney U test).

As expected, a Mann-Whitney U test on the combined sample (Sell condition: N = 84, Take condition: N = 76) showed increases in value did not extend to market estimations (Sell condition median = $7.00, Take condition median = $6.50, U = 3074.00, p = 0.69, Fig. 3A); But, again, WTA values were significantly higher for participants told explicitly to “sell” their good than those asked to “take for” (Sell condition median = $6.00, Take condition median = $5.00, U = 1955.00, p < 0.001, Fig. 3B).

**Fig 4:**
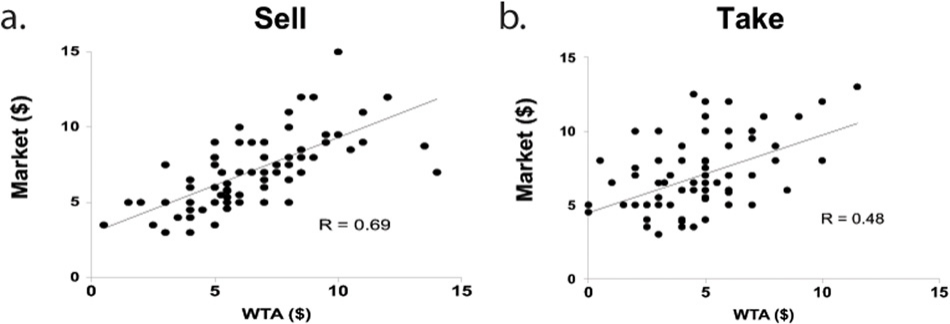
Relationship of WTA to Market Estimations. a) Correlation of WTA prices compared to participants Market estimations for participant explicitly asked to “sell” (Sell), significant at p < 0.0001; b) Correlation of WTA prices compared to participants market estimations for participant explicitly asked to “take for” (Take), significant at p < 0.0001; Fischer r—z for the difference in correlations showed a significant difference between the correlations, significant at p < 0.05.

Weaver and Frederick (2012) suggested that market prices act as a salient reference point and that sellers raise their WTA values to be more in line with the market [8]. If the word “sell” makes participants look to their market estimations as the salient reference, and bring their WTA more in line with it, we can make two predictions. First, the difference between WTA and market estimations should be smaller in the Sell condition than in the Take condition. Second, there should be a stronger correlation between WTA values and market estimations in the Sell condition than in the Take condition. Indeed, we observed both of these results. A Mann-Whitney U test revealed that the difference between WTA values and market estimations was significantly smaller for the Sell condition compared to the Take condition (Sell condition median difference = $0.00, Take condition median = -$2.00, U = 2001.5, p < 0.001). Additionally, although WTA was significantly correlated with market estimation in both the Sell and Take conditions (Sell condition: r = 0.69, p < 0.0001; Take condition: r = 0.48, p < 0.0001, Fig. 4), this correlation was significantly stronger in the Sell compared to the Take conditions (Fischer r—z transformation, z = 2.01, p < 0.05).

## Discussion

We show that subtle instructional wording affects participants’ valuations of a good traded in a market transaction. Participants asked to “sell” a good indicated higher WTA values than participants asked to “take” money for the same good. Furthermore, the wording selectively affected participants’ subjective values and did not generalize to their perceptions of market values. These findings lie in contrast to the notion that endowment status is the sole factor driving the difference between WTA and WTP values [1, 2, 17].

The stronger correlation between WTA and market estimates observed in the Sell compared to the Take conditions is consistent with findings put forth by Weaver and Frederick (2012) [8]. These authors show that sellers bring their WTA to be more in line with market values when market values largely exceed participants’ subjective valuations of the traded good. We extend these previous findings in two ways. First, our results show that the effect exists even when no explicit market value is provided, as our participants came into the transaction with a market value in mind that was irrespective of experimental condition. Second, our results show that asking a person to “sell” their item pushes them to more closely anchor their WTA value to their belief about the market value.

We show that subtle, seemingly intuitive, and ecologically abundant phrasing is enough to alter consumer valuations. It is likely that this effect of wording is an implicit activation of some mental representation of behaviors associated with the word “sell”, as all the information about the task and the good is identical in the Sell and Take conditions: both conditions are asked to relinquish their good for money. Such phrasing may change participants’ cognitive perspective of the task (for review see [16]) such that their behavior coincides with the construct of a ‘seller”. Indeed, Carmon and Ariely (2000) showed that gaps in WTA and WTP can be attributed to changes in how buyers and sellers view the task such that different aspects of the trade become more salient dependent on the participant’s role [19].

More generally, these results support recent process-based theories of WTA valuations and the endowment effect [19–22]. Johnson, Häubl, and Keinan (2007) suggested that participants query information about the good. They found that sellers retrieve positive aspects of the good earlier in their query than buyers and generate fewer negative aspects than buyers. Importantly, when sellers were asked to list negative aspects first, they no longer established higher WTA values in a market transaction [20]. Pachur and Scheibehenne (2012) go a step further to show differences in external information search, as well [22]. Our findings fit within these process-oriented theories in that those participants told to “sell” look to the market value when establishing their WTA. We conjecture that the word “sell” puts participants in a “selling state of mind” [16], pushing them to assess the difference between their subjective value and their belief of the market value.

Implicit to our argument that sellers are pushed into a selling state of mind is the possibility that the word “sell” frames the transaction as a loss. With that possibility, an alternative explanation for our findings could be that asking how much someone will “take” may in fact not be neutral; but instead may drive down the WTA values, perhaps by changing the frame of the transaction to a gain and circumventing the need to compensate for the loss of the good. In fact, Tversky and Kahneman (1981) showed, with their Asian disease problem, that they could easily shift the frame of a scenario from a perceived loss to a perceived gain with a simple change in wording [23]. We contend, however, that this possibility does not detract from our principle conclusion that language in instructions influences WTA values beyond that of endowment status. An interesting future direction would be to compare WTA values in an endowed condition that was asked to “sell” and a condition not endowed and asked to “sell” (a broker role, perhaps, in which one participant is selling an item for another participant) to flesh out whether the word alone is enough to drive valuations. Also, this study only investigated the WTA side of the WTA-WTP disparity in experimental market transactions. Future investigations determining whether this language effect holds when the participant is a buyer would be beneficial to our understanding of the WTA-WTP disparity. It would also be important to investigate whether these effects are robust to repeated transactions, an arguably more realistic market scenario.

## Supporting Information

S1 Dataset Data obtained from all participants in experiments 1 and 2. (XLSX)

S1 Instructions Instructions provided to the participants at the beginning of the experiment. (PDF)
