# Supplementary material for: “Selling” Value: The Influence of Language on Willingness-to-Accept
Source: PLoS One. 2015 Mar 30;10(3):e0120292. doi: 10.1371/journal.pone.0120292 (PMC4379148; doi:10.1371/journal.pone.0120292)
Supplement: S1 Instructions — (PDF) [file pone.0120292.s002.pdf]

S1 Instructions

Instructions: Sell Condition

In the following questions we are going to be referring to the pens set you just received. You are the owner of those pens now.

For this question, the experimenter will be asking you a question regarding the pens you just received. This person wants to know which you would rather have: the set of pens or an amount of money they will offer to you. At the bottom of the page is the list of possible offers they will make for the pens. What is the lowest amount of money that you would sell your pens for, a.k.a. your bottom line?

Note: It is important that you indicate the real amount that the pens are worth to you. After you have indicated, the experimenter will draw a dollar amount randomly and this will count as the "final offer". If the price drawn is less than your "bottom line" you have to reject the offer and take the pens. If the amount is at or higher than you bottom line, you accept the offer and get the money instead of the pens.

For example: You name your bottom line at $5.00 and the offer price of $6.50 is generated. Since the offer is above your bottom line, you will sell back the pens. If, say, 4.50 is drawn, this amount is below your bottom line. So, you will keep the pens and reject the money.

Instructions: Take Condition

In the following questions we are going to be referring to the pens set you just received. You are the owner of those pens now.

For this question, the experimenter will be asking you a question regarding the pens you just received. This person wants to know which you would rather have: the set of pens or an amount of money they will offer to you. At the bottom of the page is the list of possible offers they will make for the pens. What is the lowest amount of money that you would take for the pens, a.k.a. your bottom line?

Note: It is important that you indicate the real amount that the pens are worth to you. After you have indicated, the experimenter will draw a dollar amount randomly and this will count as the "final offer". If the price drawn is less than your "bottom line" you have to reject the offer and take the pens. If the amount is higher than you bottom line, you accept the offer and get the money instead of the pens.

For example: You name your bottom line at $5.00 and the offer price of $6.50 is generated. Since the offer is above your bottom line, you will take the money for the pens. If, say, 4.50 is drawn, this amount is below your bottom line. So, you will keep the pens and reject the money.
